# Correction

**DOI:** 10.1080/22221751.2026.2657186

**Published:** 2026-04-12

**Article title:** Emergence of the novel PA-D27G mutation conferring reduced baloxavir susceptibility in influenza A viruses circulating in China, 2018–2025

**Authors:** Sun B., Sun Y., Wang H., Tang X., Tang W., Mi Y., Shen Z., Xue Q., Lu Y., Zhao X., Ai J., Lu J. and Zhang W.

**Journal:** *Emerging Microbes & Infections*

Volume 15 Issue 1

**DOI:** https://doi.org/10.1080/22221751.2026.2620222

When this article was first published online, part label Figure 4C was incorrect. The correct part (Figure 4) is shown below and has now been updated in the online version.

**Figure 4.**
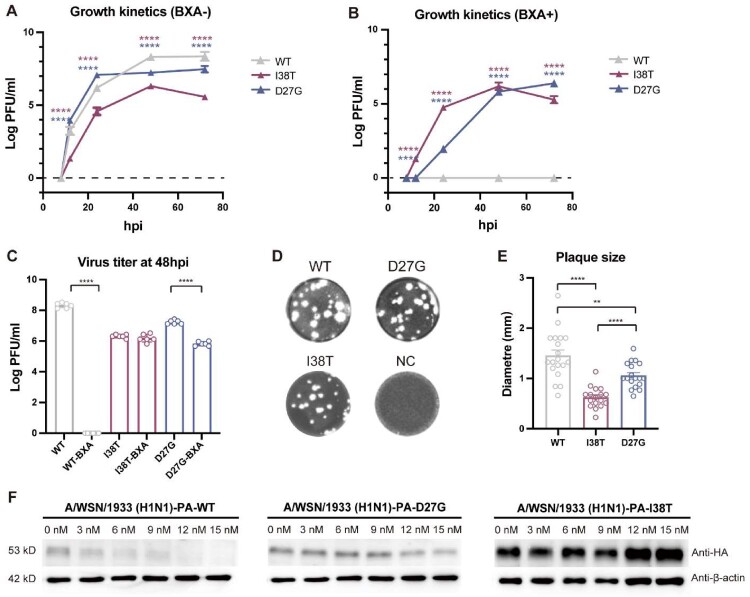
Evaluation of viral fitness of D27G in the MDCK based on A/WSN/1933 (H1N1). (A) Growth kinetics of three strains without BXA. (B) Growth kinetics of three strains with BXA. (C) Virus yield inhibition assay at 48 hpi. (D-E) Plaque assay and quantification of plaque size. (F) A/WSN/1933 (H1N1) minireplicon assays with 0–15 nM BXA. * denotes a P-value < 0.05, ** denotes a Pvalue < 0.01, *** denotes a P-value < 0.001, and **** denotes a P-value < 0.0001. Multiple t-tests were performed with false discovery rate (FDR) correction using the Benjamini – Krieger – Yekutieli method.

